# Comparative hard x-ray tomography for virtual histology of zebrafish larva, human tooth cementum, and porcine nerve

**DOI:** 10.1117/1.JMI.9.3.031507

**Published:** 2022-03-31

**Authors:** Alexandra Migga, Georg Schulz, Griffin Rodgers, Melissa Osterwalder, Christine Tanner, Holger Blank, Iwan Jerjen, Phil Salmon, William Twengström, Mario Scheel, Timm Weitkamp, Christian M. Schlepütz, Jan S. Bolten, Jörg Huwyler, Gerhard Hotz, Srinivas Madduri, Bert Müller

**Affiliations:** a University of Basel, Biomaterials Science Center, Department of Biomedical Engineering, Allschwil, Switzerland; b University of Basel, Biomaterials Science Center, Department of Clinical Research, Basel, Switzerland; c University of Basel, Core Facility Micro- and Nanotomography, Department of Biomedical Engineering, Allschwil, Switzerland; d Carl Zeiss Microscopy GmbH, Oberkochen, Germany; e Gloor Instruments AG, Kloten, Switzerland; f Bruker Micro-CT, Kontich, Belgium; g Exciscope AB, Kista, Sweden; h Synchrotron SOLEIL, Gif-sur-Yvette, France; i Paul Scherrer Institut, Swiss Light Source, Villigen, Switzerland; j University of Basel, Pharmaceutical Technology, Department of Pharmaceutical Sciences, Basel, Switzerland; k Natural History Museum of Basel, Anthropological Collection, Basel, Switzerland; l University of Basel, Integrative Prehistory and Archaeological Science, Basel, Switzerland; m University of Geneva, Department of Surgery, Geneva, Switzerland; n University Hospital Basel, Department of Plastic, Reconstructive, Aesthetic and Hand Surgery, Basel, Switzerland

**Keywords:** phase-contrast tomography, image registration, x-ray microscopy, phase retrieval, tooth cementum, porcine nerve, zebrafish larvae, spatial and density resolution

## Abstract

**Purpose:**

Synchrotron radiation-based tomography yields microanatomical features in human and animal tissues without physical slicing. Recent advances in instrumentation have made laboratory-based phase tomography feasible. We compared the performance of three cutting-edge laboratory systems benchmarked by synchrotron radiation-based tomography for three specimens. As an additional criterion, the user-friendliness of the three microtomography systems was considered.

**Approach:**

The three tomography systems—SkyScan 2214 (Bruker-microCT, Kontich, Belgium), Exciscope prototype (Stockholm, Sweden), and Xradia 620 Versa (Zeiss, Oberkochen, Germany)—were given 36 h to measure three medically relevant specimens, namely, zebrafish larva, archaeological human tooth, and porcine nerve. The obtained datasets were registered to the benchmark synchrotron radiation-based tomography from the same specimens and selected ones to the SkyScan 1275 and phoenix nanotom m^®^ laboratory systems to characterize development over the last decade.

**Results:**

Next-generation laboratory-based microtomography almost reached the quality achieved by synchrotron-radiation facilities with respect to spatial and density resolution, as indicated by the visualization of the medically relevant microanatomical features. The SkyScan 2214 system and the Exciscope prototype demonstrated the complementarity of phase information by imaging the eyes of the zebrafish larva. The 3-μm thin annual layers in the tooth cementum were identified using Xradia 620 Versa.

**Conclusions:**

SkyScan 2214 was the simplest system and was well-suited to visualizing the wealth of anatomical features in the zebrafish larva. Data from the Exciscope prototype with the high photon flux from the liquid metal source showed the spiral nature of the myelin sheaths in the porcine nerve. Xradia 620 Versa, with detector optics as typically installed for synchrotron tomography beamlines, enabled the three-dimensional visualization of the zebrafish larva with comparable quality to the synchrotron data and the annual layers in the tooth cementum.

## 1 Introduction

Hard x-ray microtomography is a three-dimensional (3D) imaging technique that allows for the quantitative evaluation of microstructures in post mortem tissues.^1^ Advanced instrumentation is applied for myriad scientific purposes, including the anatomical analysis of zebrafish larvae,^2^ the characterization of annual layers in cementum of human teeth,^3^ and the visualization of paraffin-embedded nerves.^4^ Traditionally, the highest density and spatial resolutions have been achievable at synchrotron radiation facilities. However, this unique instrumentation only offers limited beam times based on successful applications or extra payments. Laboratory-based systems have been improved substantially by incorporating improved x-ray sources, phase-contrast capabilities, and x-ray detector optics.^5^ With the increasing number of such systems on the market, a detailed comparison is needed to understand the performance of these next-generation scanners with respect to other available laboratory systems and dedicated microtomography beamlines at synchrotron radiation facilities. To this end, the performances of three cutting-edge laboratory-based tomography systems, employing absorption and phase-contrast modes, were compared for the above-mentioned scientific applications. The common volumes extracted from datasets were three-dimensionally registered to synchrotron radiation-based micro computed tomography from the TOMCAT beamline at the Swiss Light Source (SLS) [Paul Scherrer Institute (PSI), Villigen, Switzerland] or the ANATOMIX beamline at the Synchrotron SOLEIL (Gif-Sur-Yvette, France) and then evaluated with respect to spatial resolution and contrast. In addition, the user-friendliness of the three next-generation scanners was appraised by a single novice.

### 1.1 Laboratory-Based Phase-Contrast X-Ray Tomography

Conventional x-ray tomography used in medicine relies on absorption contrast, which is very suitable for imaging hard tissues. Soft tissue imaging usually requires appropriate staining. As an alternative, one can take advantage of phase contrast modes to visualize tissues consisting of light elements together with hard tissue components, including teeth, bone, and plaque, because of the linear dependence of the phase shift on the electron density.^6^ For attenuation-contrast x-ray tomography, it is especially demanding, since x-ray attenuation versus atomic number exhibits a power law with an exponent between 3 and 4. As the x-ray beam passes through condensed matter, it exhibits both absorption, and with sufficient beam coherence, a phase shift.^7^ For soft tissues, the linear absorption coefficient is three orders of magnitude lower than the related coefficient for the phase shift.^8^ Thus, for the majority of medically relevant hard x-ray images of tissues in health and disease, phase-contrast methods are preferred.^8^ Several phase tomography approaches have been evaluated for soft tissue imaging.^6^^,^^8^^–^^12^ Single-distance propagation-based approaches are often the simplest to implement and generally offer the best spatial resolution. Therefore, these systems are frequently used with micro- and nanotomography beamlines at synchrotron radiation facilities and are implemented in sophisticated laboratory-based microtomography systems.

High-resolution microtomography is more and more often referred to as “virtual histology,” because it extends the anatomical information from conventional histological sections to the third dimension.^13^^,^^14^ Virtual histology yields anatomical information without physical slicing. The spatial resolution is roughly equal in the three orthogonal directions—a distinct feature compared to serial sectioning and essential for the anatomical context, e.g., nervous tissue, as well as simple and fast data acquirement for much larger samples.^5^^,^^13^ Using nanoholotomography, one can even reach a spatial resolution beyond the optical limits given by the optical means employed to image the histological slices.^15^ Such measurements, however, suffer from limited access to synchrotron radiation facilities, since the purchase of beamtime is only common for industrial research, and a research proposal can only be submitted a few times per year, which leads to substantial delays and a focus on a smaller number of priority samples. As an alternative, several research teams use virtual histology based on laboratory-based microtomography systems. The obtained results, however, are generally compromised with respect to data from synchrotron radiation-based systems. The gap between laboratory- and synchrotron radiation-based tomography data, clearly obvious a decade ago,^16^ is becoming narrower and narrower (see e.g., see Refs. 17 and 18) with only minor differences in image quality.^5^^,^^13^^,^^19^ These advances in laboratory-based approaches motivated our team to evaluate cutting-edge instrumentation with the goal to directly compare the tomographic imaging of selected, medically relevant scientific questions related to the cellular anatomy of zebrafish larvae, to the annual layers in human tooth cementum, and to the 3D representation of paraffin-embedded porcine nerves. The acquisition of the necessary radiographs from the three selected specimens, and their reconstruction, was restricted to a period of 36 h per advanced instrument, to guarantee comparability and to have a reasonable timeframe for future experiments. It should be noted that while longer experiments could yield substantially better deliverables, the 36-h period was selected as tradeoff between standard user experience and the manufacturers’ requests. As a benchmark, the three specimens were imaged, prior to measurements with the advanced instrumentation, at the tomography setups of the TOMCAT (SLS, PSI, Villigen, Switzerland) or ANATOMIX (Synchrotron SOLEIL, Gif-sur-Yvette, France) beamlines. To validate progress in imaging the three selected specimens, the laboratory-based systems SkyScan 1275 (Bruker microCT, Kontich, Belgium) and nanotom m (Waygate Technologies, phoenix|x-ray, Wunstorf, Germany) available at the core facility of the University of Basel were included in the comparison. The four to six datasets per specimen were three-dimensionally registered to segment the common volume for a qualitative and quantitative comparison of image quality.

### 1.2 Zebrafish Larvae—A Versatile Biomedical Research Model

The zebrafish larva is a well-established animal for in vivo biomedical research. This rather basic vertebrate model offers an outstanding balance between relevant physiology and accessibility regarding ethical context, a rapid and effective life-cycle, and husbandry, as well as an attractive similarity to the human genome.^20^ Therefore, the zebrafish larva finds numerous applications, including studies in pathological conditions such as kidney injury^21^ and treatment such as transplantation,^22^ to name a few. High-resolution hard x-ray tomography was used to examine the single organ-centered anatomy of zebrafish heart^23^ and muscles,^24^ as well as nanoparticle distribution.^25^ In a recent study, synchrotron radiation was applied in whole-organism histotomography, thereby enabling the extraction of cellular architectures.^26^ This study promises a broader understanding of anatomy and corresponding physiology and pathophysiology. Previously, we showed that more than 50% of anatomical features identified by synchrotron radiation-based microcomputed tomography (SRμCT) can also be identified with standard laboratory-based tomography systems.^27^ We can, therefore, expect that the cutting-edge laboratory-based systems will provide images comparable to the tomography setups at synchrotron radiation facilities. Such a level of success implies the possibility to easily perform large experimental series of high-resolution imaging fundamental in zebrafish larva-based research activities.

### 1.3 Tooth Root Cementum—A Lifelong Growing, Mineralized Tissue

Tooth cementum is a mineralized tissue that exists in vertebra teeth and covers the entire surface of the tooth root, belonging functionally to its anchor, the periodontium. In contrast to bone, this avascular complex is independent of regular remodeling, and thus it expands over a lifetime with location-dependent growth rates.^28^^,^^29^ For humans, the homogeneous structure reveals about 3-μm thin incremental layers, as originally found in optical micrographs of thin tooth slices.^30^ These incremental layers were recently detected using synchrotron radiation-based microtomography.^3^ Our team is currently evaluating data for entire teeth collected during a beamtime session at the ANATOMIX beamline in February 2021. The related analysis pipeline is presented in a recent paper.^31^ Incremental layers are seasonally deposited, similar to the well-known layers in a tree trunk. Thus, a pair of layers, consisting of dark and bright structures, represents one year.^32^ Resulting predictions of the season of death based on these layers^33^ indicates tooth cementum as a tissue highly valuable for anthropology^32^ and forensics.^34^ Layer thickness is influenced by a number of factors, including hormonal changes in pregnancy and stress events, such as pathologies as well as nutrition, as examined profoundly in recent studies.^33^^,^^35^ Nonetheless, cervical acellular extrinsic fiber cementum provides the steadiest growth rate in terms of conserving layer thickness.^28^^,^^29^^,^^32^ Unfortunately, counting these layers is error-prone and observer-dependent, leading most commonly to an underestimation of age despite high-resolution imaging methods.^36^^,^^37^ Further investigation is therefore desired and suggested in archeological samples with corresponding life history.^32^^,^^37^ For these unique ancient samples, tomographic imaging should be favored to conventional microscopy, the latter requiring physical slicing.^3^ In this study, we show to what extent the incremental layers can be detected by means of laboratory-based tomography setups. It can be reasonably assumed that owing to the developments in x-ray source and detector technology for the advanced instrumentation, incremental layers could come to light.

### 1.4 Nerves—Clinical Application of Hard X-Ray Tomography-Controlled Products

Nerves show slow self-healing and some reinnervation potential after damage up to a minimum twelve months post-injury following a degenerative phase. Surgical treatment might be needed, especially in neuronal endplate involvement, i.e., neurorrhaphy or even grafting in the case of potential tension by end-to-end suturing.^38^ Still, more than one-quarter of patients do not regain motor function^39^ without substantial improvement over the last two decades.^38^^,^^39^ Additionally, the quantification of nerve damage, which currently relies on conventional histology, assists in understanding the pathomechanisms, diagnostics, and therapy for multiple sclerosis^40^ and vasculitis, including Wegener’s polyangiitis.^41^ Microtomography with resolution down to the sub-cellular level has been proposed as a tool for nerve imaging and further investigations into nerve regeneration.^4^^,^^42^ In contrast to histology, phase-contrast microtomography of myelinated nerves provides visualization and the quantification of the microanatomy of nerves and may lead to profound physiological comprehension.^43^^,^^44^ We hypothesize that advanced laboratory instrumentation will lead to substantial improvements in microanatomical feature visibility compared to established laboratory-based tomography systems.^4^^,^^42^^,^^45^

### 1.5 Assessment of User-Friendliness

A satisfying experience with a purchased product strongly depends on a subjective evaluation of usability rather than purely objective criteria such as effectiveness and efficiency.^46^ Assessments of software user-friendliness have thus been well-established since the 1980s through the use of standard questionnaires, including the system usability scale.^46^ Crucial criteria appraised by the analysis of comments on tested systems include easy handling and intuitive design.^47^ Unintuitive systems run the risk of malfunction, leading to reduced overall performance of technology, and may even be hazardous in a medical context.^48^ Therefore, we included the user-friendliness of advanced laboratory systems as a valid purchasing criterion for next-generation systems. We integrated four criteria to appraise novice user experience in cutting-edge setups: (i) intuitive interface, (ii) structural organization, (iii) efficiency and effectiveness, and (iv) reliability. These areas were scored by a single beginner-level user who participated in the test measurements.

## 2 Materials and Methods

### 2.1 Sample Preparation

#### 2.1.1 Preparation of three-day-old paraffin-embedded zebrafish larvae

Three-day-old zebrafish larvae were euthanized using tricaine methanesulfonate containing 0.612-mM trisamino-methane. Subsequently, the larvae were fixated in 4% paraformaldehyde at room temperature before refrigerating the samples at a temperature of 4°C for storage. These fixed larvae were dehydrated in a dilution series of ethanol from 25% via 50% and 70% to above 99.5% in time steps of 15 min. Then, the dehydrated zebrafish larvae were washed twice in xylene, >98%, Carl Roth, Switzerland, and subsequently transferred into liquid paraffin at a temperature of 68°C (Leica Microsystems, Wetzlar, Hesse, Germany). After cooling, metal punches with inner diameters of 2.8 or 3.6 mm were used to cut out the embedded specimens to obtain cylinders for the imaging tasks. For the synchrotron radiation-based experiments, the paraffin cylinders were further manually trimmed to remove excess paraffin.

#### 2.1.2 Selection of archaeological human tooth

The selected premolar tooth from the maxilla of a woman who died at the age of 36 comes from the reference skeleton series Basel-Spitalfriedhof, which is archived at the Natural History Museum Basel, Switzerland.

The skeletons were exhumed in 1988 and 1989 from the cemetery of the former Basel City Hospital. Based on historical sources, it was possible to identify the skeletons of former hospital patients, who died between 1845 and 1868. In addition, further information on social and geographical origin, information on living and working situations, medical histories, etc., are available. This detailed historical information, combined with corresponding skeletons from the 19th century, is unique worldwide and allows, e.g., the reconstruction of biographies taking into account biological and historical sources. Furthermore, these skeletons are used for method verification and the development of future methods.

#### 2.1.3 Preparation of porcine nerves for tomographic imaging

After excision, the peripheral nerves were processed by following a standard histology protocol for formalin fixation and paraffin embedding. Briefly, the nerves were straightened by firmly tugging both ends with surgical forceps, fixed in histology-grade 4% formalin over a period of 24 h, and then dehydrated in ascending ethanol solutions. Subsequently, nerves were transferred to xylene and then perfused in a liquid paraffin-polymer mixture (Leica Paraplast, Muttenz, Switzerland).

When liquid paraffin perfusion was completed, the nerves were removed from the histological tissue processor, placed in a metal container, and left for a period of 24 h inside an oven at a temperature of 60°C. This step is important in removing air bubbles trapped inside or around the specimen, as they can potentially cause artifacts during x-ray imaging and compromise automatic data analysis. Subsequently, the specimens were thoroughly washed under flowing liquid paraffin, to remove high-absorbing particles or debris on the sample surface that would affect imaging quality. Finally, the nerves were immersed in paraffin several times while holding on one edge, until a uniform cylindric specimen was formed and then cooled down to a temperature of 4°C over a period of 15 min.

### 2.2 Data Acquisition with SkyScan 2214

The samples were mounted on a thin carbon fiber stage for high-resolution nanoCT scanning in the SkyScan 2214 system. It is noteworthy that the SkyScan 2214 setup consists of up to three cameras that can be easily exchanged by the user. The selected x-ray camera was set in a near position with a source-camera distance of ∼235  mm (for details, see Tables 1–3). Acceleration voltage was set to 40 kV for imaging the zebrafish larva, 70 kV for imaging the tooth cementum, and 30 kV for the porcine nerve bundle. A 0.5-mm thin aluminum filter was only applied for data acquisition of the human tooth. An ${\mathrm{LaB}}_{6}$-type source filament was employed for all scans; depending on the desired spatial resolution and flux, W or ${\mathrm{LaB}}_{6}$ cathodes can be implemented. The individual scans were limited to a duration of <11  h. Spot size was about 0.5  μm for zebrafish larva and tooth radiograph recording, and about 1.5  μm for nerve bundle recording. Scan pixel sizes were set to 330, 750, and 800 nm for zebrafish larva, tooth, and nerve bundle, respectively. The total numbers of recorded projections were 3001, 2118, and 2401, for zebrafish larva, tooth, and nerve fiber bundles, respectively. All scans were acquired over 360 deg, and frame averaging was employed. For the zebrafish larva and tooth imaging, two images per projection were averaged, while four images were used per nerve projection. Active ring artifact suppression by random horizontal (compensated) camera movement was done for all scans. Post-scan correction was applied^49^ to minimize thermal movement artifacts. Image reconstruction was performed by a Feldkamp-type cone-beam algorithm,^50^ using Bruker NRecon™ software with GPU acceleration and by applying Gaussian smoothing, ring artifact, and beam hardening corrections. Phase retrieval was also carried out for the acquired data.^51^

**Table 1:** Scanning parameters used in zebrafish larva microtomography.

	Nanotom m	SkyScan 1275	SkyScan 2214	Exciscope	Xradia 620 Versa	TOMCAT
Source-camera distance (mm)	600	286.0	237.5	280.0	12.6	25,012.0
Source-object distance (mm)	6.5	13.7	8.65	225.0	6.0	25,000.0
Effective voxel size (μm)	1.1	3.7	0.33	1.28	0.33	0.33
Acceleration voltage (kV)	60	15	40	40	50	(12 keV)
Beam current (μA)	310	156	116	1400	90	
Horizontal FOV (pixels)	3072	1944	4032	2048	2048	2560
Number of radiographs	720	720	3001	3600	4801	2000
Rotation steps (deg)	0.5	0.5	0.12	0.1	0.07	0.09
Exposure time (s)	9	2.5	4.7	7	8	0.2
Scan time (h)	2.5	4	10.5	7.25	14	0.15

**Table 2:** Scanning parameters used in human tooth cementum microtomography.

	SkyScan 2214	Exciscope	Xradia 620 Versa	ANATOMIX
Source-camera distance (mm)	235.2	280.0	128.55	170,050.0
Source-object distance (mm)	10.1	225.0	13.55	170,000.0
Effective voxel size (μm)	0.75	1.28	0.71	0.65
Acceleration voltage (kV)	70	70	80	(33 keV mean)
Beam current (μA)	80	1400	125	
Horizontal FOV (pixels)	4032	2048	2048	11,300
Number of radiographs	2118	3600	2501	9000
Rotation steps (deg)	0.17	0.1	0.14	0.04
Exposure time (s)	7	12	7 to 14	0.1
Scan time (h)	11	12.25	8.5	0.75

**Table 3:** Scanning parameters used in porcine nerve microtomography.

	SkyScan 1275	SkyScan 2214	Exciscope	Xradia 620 Versa	ANATOMIX
Source-camera distance (mm)	286.0	235.2	280.0	44.35	170,050.0
Source-object distance (mm)	30.2	10.8	225.0	9.35	170,000.0
Effective voxel size (μm)	8.5	0.8	1.28	0.71	0.65
Acceleration voltage (kV)	20	30	40	60	(33-keV mean)
Beam current (μA)	175	150	1400	108	
Horizontal FOV (pixels)	1944	4032	2048	2048	15,000
Number of radiographs	720	2401	3600	3201	9000
Rotation steps (deg)	0.25	0.15	0.1	0.11	0.04
Exposure time (s)	0.65	1.1	10	6.2	0.05
Scan time (h)	2.3	6.0	10.25	7.5	0.5

### 2.3 Data Acquisition with Exciscope

The three selected samples were imaged at KTH Royal Institute of Technology in Stockholm, Sweden. The setup is based on the MetalJet D2 x-ray source (Excillum AB, Kista, Sweden), the XSight Micron LC 1080 sCMOS X-ray detector (Rigaku Innovative Technologies Europe s.r.o., Czech Republic) and the URS100BCC rotation stage (Newport, California, United States). Both acquisition control and image reconstruction were done using Exciscope software (Exciscope AB, Kista, Sweden). The experimental setup had a source-object distance of 225 mm and a source-detector distance of 280 mm. Effective pixel size was set to 1.28  μm at the scintillator and 1.03  μm at the object, due to geometric magnification. Projections were acquired equiangularly over a full 360 deg rotation. The reconstruction included phase retrieval with Paganin’s method^52^ and tomographic reconstruction using the FDK method.^50^ The scan parameters for the three samples are listed in Tables 1–3.

### 2.4 Data Acquisition with Xradia 620 Versa

X-ray microscopy measurements were performed on a Zeiss Xradia 620 Versa system (Carl Zeiss X-ray Microscopy, Inc., Dublin, California, United States). Projections of the samples were recorded in a unique two-stage magnification process that included optical magnification. For the zebrafish larva data recording, we employed an acceleration voltage of 50 kV, a beam current of 90  μA, a 20× objective, 325-nm effective pixel size, an 8 s exposure time, and 4801 projections. For the human tooth we used an acceleration voltage of 80 kV, a beam current of 125  μA, a 4× objective, narrowing of the x-ray bandwidth by the LE4 filter, 710-nm effective pixel size, a rotation angle-dependent exposure time ranging from 7 to 14 s, and 2501 projections. The porcine nerve was imaged with an acceleration voltage of 60 kV, a beam current of 108  μA, a 4× objective, 709-nm effective pixel size, an exposure time of 6.2 s, and 3201 projections. The tomography datasets were reconstructed with ZEISS Scout-and-Scan Reconstructor software, using the cone-beam method for the tooth and zebrafish larva data and the OptiRecon method for the nerve data. In addition, the zebrafish larva data were reconstructed with the DeepRecon Pro reconstruction module and post-processed with the PhaseEvolve software module.

### 2.5 Data Acquisition at the TOMCAT Beamline

Synchrotron radiation-based phase-contrast x-ray microtomography measurements of zebrafish larva were acquired at the TOMCAT beamline X02DA of the SLS.^53^ The x-ray beam generated by the 2.9 T superbend magnet was monochromatized to 12 keV at a bandwidth of around 2%, using an Ru/C double multilayer monochromator. Projection images of the sample placed about 25 m from the source were converted to visible light by a 20-μm thick ${\mathrm{Lu}}_{3}{\mathrm{Al}}_{5}O_{12}:\mathrm{Ce}$ scintillator (Crytur, Turnov, Czech Republic) positioned 12-mm downstream of the sample. The image on the scintillator was magnified 20-fold by a visible-light microscope (Olympus UPLAPO 20× objective) and recorded by a pco. Edge 5.5 sCMOS camera (PCO, Germany) with a native pixel size of 6.5  μm, resulting in an effective pixel size of 0.325  μm. During the continuous 180 deg rotation of the sample (parallel beam geometry), 2000 projections with 200-ms exposure time were recorded for the reconstruction, resulting in an angular step of 0.09 deg and a scan time of 400 s. Additionally, 30 dark-field and 50 white-field images were recorded for image correction. Propagation-based phase-contrast projections were calculated from the flat field- and dark field-corrected radiographs, using Paganin’s algorithm with the parameters $\delta =10^{-7}$ and $\beta =2\;10^{-9}$.^52^ The absorption- and phase-contrast reconstructions were then computed from the corrected and phase-filtered projections, respectively, with the gridrec reconstruction algorithm.^54^

### 2.6 Data Acquisition at the ANATOMIX Beamline

Measurements at the Synchrotron SOLEIL, ANATOMIX beamline^55^ were taken with a filtered white beam at a gap of 5.5 mm from the U18 in-vacuum undulator source of the beamline. The beam was filtered with 20-μm thick Au and 100-μm thick Cu films. The resulting beam had an estimated mean photon energy of about 33 keV. The detector consisted of a 20-μm thick ${\mathrm{Lu}}_{3}{\mathrm{Al}}_{5}O_{12}:\mathrm{Ce}$ scintillator (Crytur, Turnov, Czech Republic) coupled to a scientific-grade CMOS detector (Hamamatsu Orca Flash 4.0 V2) of 2048×2048  pixels by microscope optics (Mitutoyo 10× M PLAN APO, numerical aperture 0.28) and with a magnification of 10, resulting in an effective pixel size of 0.65  μm. As the size of the nerve and the tooth were larger than the field of view, we performed an extended-field acquisition, where we acquired scans at four and three off-center positions, respectively. The neighboring radiographs were stitched together based on maximizing cross-correlation in the overlapping regions. At an electron current of 450 mA in the SOLEIL storage ring, exposure time for each of the 9000 projections per height step/ring taken over a range of 360 deg was 50 and 100 ms, respectively. The scan was taken in continuous on-the-fly mode. With the time required for the acquisition of flat and dark images, and including dead time, the overall scan time for each height step was around 30 and 45 min, respectively. Before the absorption-contrast reconstruction, the projections were filtered with a Gaussian of width σ=1.25  pixel and 0.75 pixel, respectively, to increase the contrast-to-noise ratio (CNR).^56^

### 2.7 Data Acquisition with Nanotom m^®^ and SkyScan 1275

Parameters for data acquisition of the three selected specimens, i.e., paraffin-embedded zebrafish larva and porcine nerve, using the established microtomography systems nanotom m^®^ and SkyScan 1275 are given in Tables 1 and 3.

### 2.8 Data Registration

The registration pipeline consisted of several steps. First, we found an approximate position in the SRμCT datasets corresponding to the volume imaged in the laboratory scanner. The laboratory-based volume was then manually pre-aligned with this SRμCT image region via ITK-SNAP^57^ (version 3.8.0). The images were then automatically registered with an affine or similarity transformation employing the open-source registration toolbox elastix (version 4.9).^58^^,^^59^ In the case of the zebrafish larva and tooth specimens, the foreground region was determined by semi-automatic segmentation via thresholding and morphological operations. Image registration parameters were tuned by checking the progression of the image similarity measure during registration and by visually inspecting alignment. The original laboratory-based μCT volumes were transformed to the space of the SRμCT volumes region using cubic B-spline interpolation, as this preserved image intensities far better than nearest-neighbor and linear interpolation.^60^ Registrations of the zebrafish larva, tooth cementum, and porcine nerve bundle images were performed independently by three of the authors (G.R., M.O., and C.T.). The registration parameters are listed in Table 4.

**Table 4:** Main registration parameters for the three classes of samples.

	Zebrafish larva	Tooth cementum	Pig nerve bundle
Transformation	Affine	Affine	Similarity
Image similarity measure	Normalized correlation coefficient	Normalized correlation coefficient	Advanced Mattes mutual information
Multi-resolution image pyramid with three levels	16×, 8×, 4×	4×, 2×, 1×	4×, 2×, 1×
Number of iterations	4000	2000 or 3000	3000
Number of sample points	8192	130,000 or 1,950,000	65,536

### 2.9 Identification of Anatomical Structures

Representative slices of the tomography datasets were compared in anatomy to atlas data. For the paraffin-embedded zebrafish larva, the images were compared to histological slices published in the Zebrafish Lifespan Atlas.^61^ Qualitative comparison was done for the zebrafish larva dataset, because the same region is imaged in every dataset.

For the paraffin-embedded porcine nerve, the porcine sciatic nerve model was used.^62^

## 3 Results and Discussion

Image quality was initially evaluated by visual assessment, i.e., a microanatomical description, with subsequent calculation of the spatial resolution. The representative cross-sectional virtual histology slices acquired with synchrotron radiation sources showed a wealth of anatomical features, therefore, serving as the gold standard. In Sec. 3.1, we present a comparison of the zebrafish larva and the porcine nerve alongside the results of the currently available inhouse systems nanotom m^®^ and SkyScan 1275. Sections 3.2, 3.4, and 3.6 contain the results of the next-generation laboratory systems for the three selected specimens in comparison to the synchrotron radiation-based microtomography data for the same specimens.

### 3.1 Descriptive Microanatomy of Selected Tomographic Slices

**Fig. 1:**
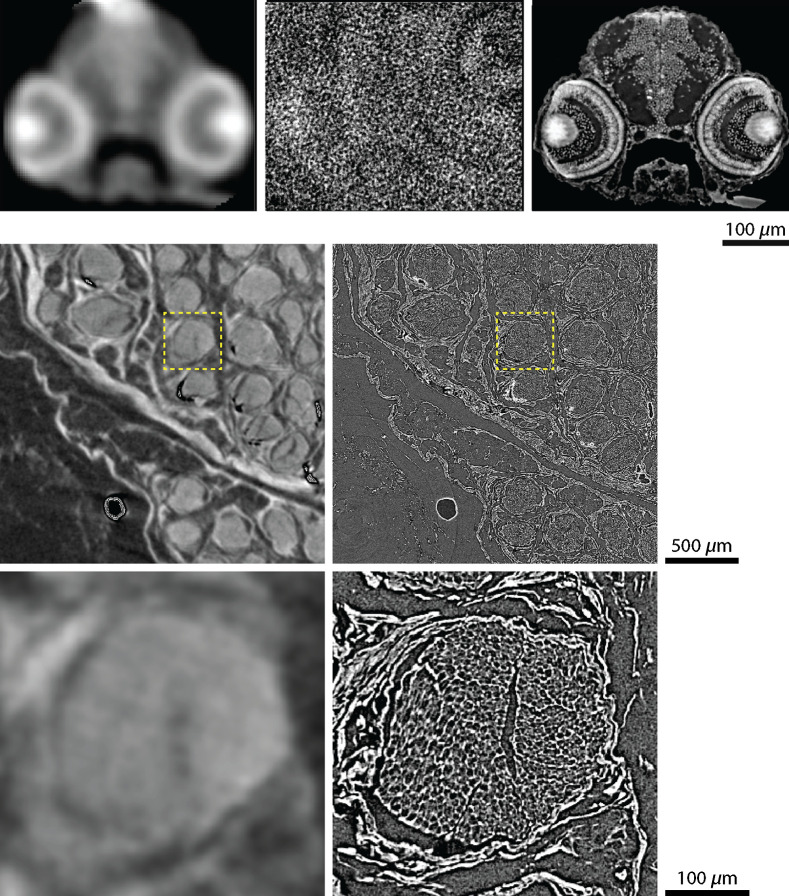
First row: corresponding virtual slices through the zebrafish larva head by means of the established laboratory-based microtomography systems SkyScan 1275 and nanotom m^®^ in comparison with data from the TOMCAT beamline (left to right), which can obviously serve as the gold standard in zebrafish larva imaging. Second row: corresponding cross-sectional slices of unprocessed, paraffin-embedded porcine nerves. Using 5-μm wide pixels, SkyScan 1275 (left) provides poorer spatial resolution in comparison to the ANATOMIX beamline (right) with 0.65-μm wide pixels and propagation-based phase contrast, obtaining anatomical features down to the sub-cellular level. The magnified views in the third row, the locations of which are indicated by the yellow-colored dashed lines, show a selected nerve fiber bundle. The image clearly demonstrates the gap between established laboratory-based systems and tomography setups at synchrotron radiation facilities.

Cross-sectional slices of the paraffin-embedded zebrafish larva head are shown in Fig. 1, top row. The data acquired at the TOMCAT beamline, image on the right, are phase-retrieved and demonstrate true single-cellular and even subcellular resolution. In particular, one can see mesencephalic and ophthalmic nuclei. Individual ocular cell layers that can be distinguished are the ganglion cells, inner plexiform, amacrine cells, the photoreceptor layer, and the retinal pigmented epithelium—apart from the distinction between the bipolar and outer plexiform layers. Mesencephalic nuclei with high electron density are separated from their surroundings; however, corresponding cartilage features around the pharynx remain low in contrast.

The established laboratory-based systems SkyScan 1275 and nanotom m^®^ (top row, left and center images of Fig. 1) do not yield sufficiently high spatial resolution or contrast, respectively. It is noteworthy that the nanotom m^®^ data barely provide meaningful images of this low-absorbing specimen, since the aluminum layer on the detection unit suppresses photons with energies below 30 keV. Therefore, only the otoliths and the overall shape are visible as shown in a recent study.^25^ The SkyScan 1275 system allows the detection of photons with an energy down to 10 keV, where even the ocular layers can be distinguished. The contrast between the denser nuclear mesencephalic region and its surroundings is satisfactory. Limited spatial resolution, however, prevents true cellular resolution.

The data from ANATOMIX beamline have revealed the characteristic anatomy of the peripheral nerve, which consists of epi-, peri- and endoneurium, as well as primary nerve fiber bundles surrounded by myelin sheaths. The latter is highlighted in the magnified view (Fig. 1, lower right, with location given by the yellow dashed box). The vasa nervorum was invisible due to a lack of contrast. The absorption-contrast SkyScan 1275 datasets (cp. second row of Fig. 1 left) allowed for visualization of the blood vessels; however, myelin sheaths could not be resolved due to insufficient spatial resolution or contrast.

### 3.2 Comparing Zebrafish Larva Imaging of Next-Generation Laboratory-Based Scanners with Tomographic Imaging Available at TOMCAT Beamline

#### 3.2.1 Performance of SkyScan 2214

**Fig. 2:**
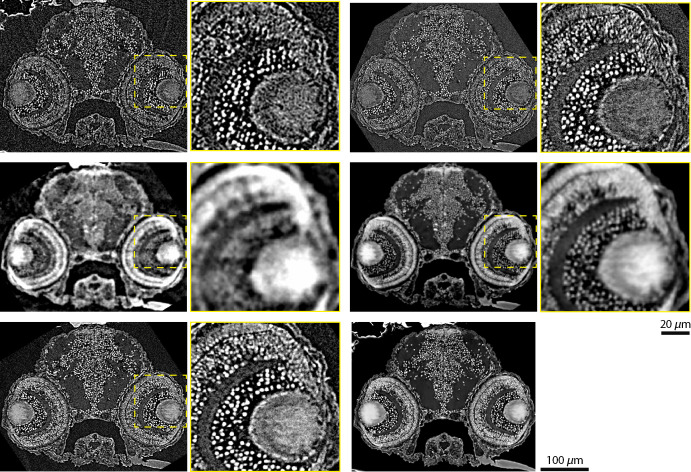
The tomographic imaging of the zebrafish larva shows comparable results between the synchrotron radiation-based and next-generation laboratory-based instrumentation. First column: corresponding virtual slices through the zebrafish larva head by means of the cutting-edge, laboratory-based microtomography SkyScan 2214 system (top row) in comparison with data acquired at Exciscope (second row) and absorption-contrast tomography images from Xradia 620 Versa (third row). The dashed yellow squares indicate the position of the enlarged views (second column). Third column: corresponding virtual slices through the head of the zebrafish larva recorded at the TOMCAT beamline; the first row shows data reconstructed without phase retrieval, and the second row shows it after Paganin phase retrieval. The dashed yellow squares indicate the position of the enlarged views (fourth column). The image in the third column and third row is obtained from the Xradia 620 Versa instrument using software including the Zeiss PhaseEvolve algorithm.

The comparison of the images in the top row of Fig. 1 with the ones in Fig. 2 elucidates the leap in evolution from established microtomography to cutting-edge systems. Even the most affordable among the three systems, namely, SkyScan 2214, allows for the resolution of the individual cells within the zebrafish larva head, as especially recognized in the images with higher magnification provided in the right column (see left part of the top row). The similarity to the gold standard data (right part of the top row) is striking. The eye region with ophthalmic cells were used for estimating spatial resolution, using the Fourier domain.^63^ Briefly, the logarithm of the squared norm of the Fourier transform was plotted as a function of the squared distance from the origin, then a linear fit allowed for the estimation of the width of a Gaussian point spread function. The instrument at the TOMCAT beamline yielded 1.3  μm, whereas SkyScan 2214 data produced 1.6  μm.

#### 3.2.2 Performance of Exciscope

The related virtual slices through the zebrafish larva shown in the left part of the second row in Fig. 2 were prepared by including phase retrieval with Paganin’s method. Therefore, the TOMCAT beamline data given in the right part of the second row of Fig. 2 exhibit modified contrast with respect to the images in the top row of Fig. 2. In addition, the spatial resolution has been compromised. Cellular resolution was mostly preserved, although several anatomical features of the eye vanished, owing to noise level. This behavior is even more pronounced in the data for Exciscope, most probably because of the less appropriate detector optics. The individual cells can hardly be recognized; hence, the Exciscope system—at least in the configuration used herein—cannot resolve the cells within a paraffin-embedded zebrafish larva. The eye region with the ophthalmic cells (magnified view) was also used for estimating spatial resolution within the phase-retrieved datasets.^63^ Here, the phase retrieved data from the TOMCAT beamline yielded 1.8  μm and the Exciscope 4.3  μm.

#### 3.2.3 Performance of Xradia 620 Versa

The selected virtual slice through the zebrafish larva obtained with the Xradia 620 Versa system was recorded in the absorption contrast mode (see left part of third row in Fig. 2). Xradia 620 Versa resembles data from the TOMCAT beamline, see top row right part, best, most probably because of the same optical components in both detection systems. Some edge enhancement is visible in these two datasets, e.g., the feature in the lower-right corner. The eye region with the ophthalmic cells used to estimate spatial resolution^63^ gave rise to a spatial resolution of 1.6  μm, slightly above the value mentioned for the tomography setup at TOMCAT beamline (1.3  μm).

The Xradia 620 Versa setup could only provide reconstructions based on absorption contrast (status March 2021). The manufacturer, however, has stated that a feature for image enhancement using phase contrast will be available soon. In August 2021, the reconstruction module DeepRecon Pro was presented.^64^ In this reconstruction, the image contrast with respect to propagation-based phase-contrast effects was enhanced using the PhaseEvolve module with parameters: fringe width 5.24 and fringe strength 12 (right image in bottom row of Fig. 2). Depending on the chosen parameters, the result is comparable to the single-distance phase-retrieved data set, shown above in the second row of Fig. 2.

### 3.3 Zebrafish Larva—Full-Specimen Imaging in Pre-Medical Studies

Concerning the detection of anatomical features Xradia 620 Versa showed the highest resolution, i.e., down to single nuclei; the contrast in the provided images was further improved with the software PhaseEvolve. SkyScan 2214 produced a likewise high-quality dataset with a slight increase in noise. Distinction of cells was impossible with Exciscope, but it nonetheless showed satisfactory layer separation within the zebrafish larva eye. It is noteworthy that this was achieved in unstained samples, whereas, previously, staining has been widely used with laboratory instrumentation.^23^ A detailed comparison of the three x-ray microscopes was impossible, as at the time of our measurement, i.e., March 2021, the DeepRecon Pro and PhaseEvolve software modules were not available yet in Europe for the Xradia 620 Versa, SkyScan 2214 data measurements had to be repeated, and the Exciscope prototype only provided detector optics with an effective pixel size of 1.3  μm. Following visual inspection, however, advances in table-top μCT systems, in comparison to the established systems in our lab, were evident, leading to an image quality close to that of SRμCT, although at longer scan times.

Recent studies of zebrafish larvae, e.g., their complete histological phenotyping^26^ and the determination of nanoparticle distribution,^25^ have relied on SRμCT. Based on the present results with cutting-edge μCT systems, future studies of the zebrafish larva in a laboratory setting are planned. Moreover, with the attractive homology to humans, as underlined by exemplary drug target conservation,^65^ further investigations of pharmacological and phenomics studies with high-resolution imaging of organs and whole zebrafish larva will be led by cellular resolution in an accessible setup for fast-feedback and follow-up imaging. This volumetric imaging provides a 3D context—in contrast to conventional histology serving as an alternative for pharmacological studies. Future zebrafish larva studies will include imaging the progression of kidney diseases and the renal regeneration with the goal to identify therapeutics that enhance human renal regeneration.^21^

### 3.4 Comparing the Imaging of Annual Layers in Human Tooth Cementum by Applying Next-Generation Laboratory-Based Scanners and ANATOMIX Beamline Microtomography

**Fig. 3:**
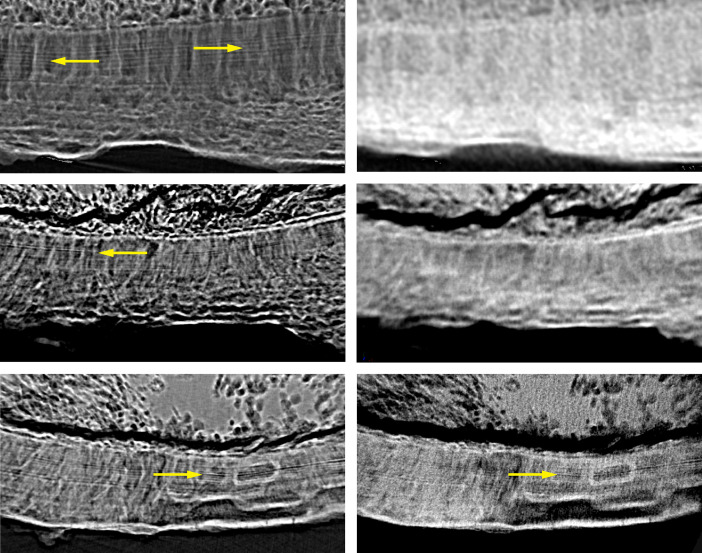
Selected regions of interest from the human tooth cementum acquired at the ANATOMIX beamline (images in the left column) and registered data from SkyScan 2214 (right image, top row), from Exciscope (right column, middle image), and from Xradia 620 Versa (right column, bottom image). The registered data are represented in a special 3D fashion: 100 slices were rendered using the sum along ray tool from VGStudio MAX to improve the visibility of the incremental layers (see yellow arrows).^31^ Annual layers are visible not only in data acquired at the synchrotron radiation facility, but also in the dataset gathered at the cutting-edge laboratory-based Xradia 620 Versa system. The width of each image equals about 600  μm.

**Fig. 4:**
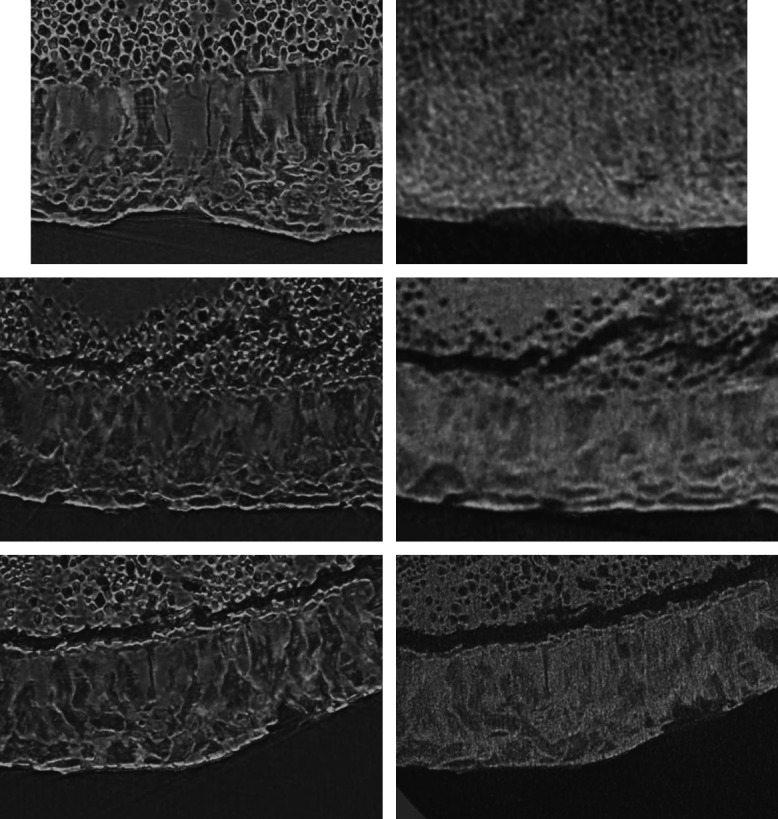
A still image of Video 1 showing the annual layers are detectable in the movies in the left column and at the one on the bottom in the right column: Scroll-through 160 cross-sectional virtual histology slices, prepared by the software VGStudio MAX, of the human tooth obtained at the ANATOMIX beamline (images in the left column) and corresponding local scans by means of SkyScan 2214 without phase retrieval (right column, top row), of Exciscope with a priori phase retrieval (right column, middle row), and of Xradia 620 Versa without phase retrieval (right column, bottom row). The width of each box equals about 600  μm (Video 1, MP4, 11639 KB [URL: https://doi.org/10.1117/1.JMI.9.3.031507.1]).

Applying mosaic-style acquisition,^66^ the entire human tooth cementum was made visible by means of single-distance phase-contrast tomography. This approach avoided the presence of artifacts known from local scans with true micrometer resolution when objects with centimeter diameters were studied. Thus, local tomography data from the three selected next-generation, laboratory-based tomography systems could be registered to the large dataset from the ANATOMIX beamline. This approach was helpful, since local tomography at pre-defined positions could not be guaranteed. Therefore, the data shown in Figs. 3 and 4 do not cover the same regions of the human tooth, but they have been always registered to the same ANATOMIX dataset. Consequently, the comparability of tomography data from the cutting-edge instrumentation is definitely provided.

#### 3.4.1 Performance of SkyScan 2214

The part of the human tooth shown in the first row of Fig. 3 is a superb choice, because many annual layers can be traced from left to right within data from the ANATOMIX beamline (see left image). As found previously,^3^ the 3-μm thin annual layers are much clearer in the low-absorbing parts of the tooth cementum. The SkyScan 2214 setup, however, only shows contrast between the lower and higher x-ray-absorbing regions in the tooth cementum, while the annual layers remain invisible. This observation is also supported by Fig. 4 (first row), when scrolling through the 160 selected virtual slices obtained from the datasets of the two tomography systems.

#### 3.4.2 Performance of Exciscope

The images in Fig. 3, middle row shows a part of the human tooth with the following anatomical features. In the upper part, one finds dentin with a crack of characteristic morphology. In the middle, tooth cementum with the annual layers is found. The lower part in black represents the surrounding air. Whereas the annual layers are easily recognized within the data from the ANATOMIX beamline, indicated by the yellow-colored arrow, Exciscope instrumentation in the configuration used could not resolve these anatomical features with an average thickness of only 3  μm. An objective with higher resolution, however, might master this deficiency. These findings are further elucidated by Fig. 4. It is noteworthy that in Fig. 4, middle part explicitly shows that the thin lines, usually termed incremental lines, are actual 3D layers, given by the continuity of the visible lines scrolling through the 160 slices of the synchrotron radiation-based dataset.

#### 3.4.3 Performance of Xradia 620 Versa

The bottom rows in Figs. 3 and 4 demonstrate that annual layers can also be made visible with a laboratory-based system. Although Xradia 620 Versa does not reach the resolution of the synchrotron radiation-based setup, this result is nevertheless promising and could even be further improved by substantially increasing acquisition time. It is noteworthy that the manufacturers had only 36 h to image the three selected samples, and the flux from the laboratory sources is orders of magnitude less than that of the synchrotron radiation facilities.

### 3.5 Human Tooth Cementum—Requirement for Non-Destructive Imaging Techniques in Unique Samples

To date, incremental layers may only be imaged to a satisfying extent at synchrotron radiation facilities^37^ or by means of conventional optical microscopy of thin sections. ^32^^,^^34^^,^^36^ A laboratory-based μCT was suggested by Mani-Caplazi et al.^3^ based on physically sliced samples and a close source-sample position. In the current study, we showed that advanced laboratory instrumentation generates great potential for facilitating high-resolution incremental layer imaging in a non-destructive manner. Barely identifiable lines in tooth cementum obtained with Xradia 620 Versa were enhanced by an oriented projection of 100 slices.^31^ High-resolution, 3D imaging of human tooth cementum, as evidenced in the SRμCT or μCT, provides a sufficient anatomical context to show that the thin lines actually correspond to curved 3D layers,^67^ which we showed in scroll-through Fig. 4, where those lines were continuous.

Our findings demonstrate great potential for laboratory sources in ongoing research into tooth cementum annulation (TCA). This phenomenon has been studied in humans for around four decades^32^; however, there is—as of yet—neither a coherent explanation nor a standard protocol for tooth selection, preparation, and layer counting, and yet many advances have been made.^32^^,^^34^ Laboratory-based μCT may benefit region selection applied a priori to SRμCT^3^ or conventional microscopy, where samples are cut irreversibly into about 100-μm thin slices.^36^ Furthermore, the promising results offered by Xradia 620 Versa indicate that laboratory-based μCT alone could be used to quantify these layers, e.g., in large-scale studies of archeological samples with recorded history, to standardize age estimation or examine the factors influencing layer growth. This approach might be extended to animal studies.

### 3.6 Comparing the Three-Dimensional Imaging of the Spiral Organization in a Paraffin-Embedded Porcine Nerve by Applying the Next-Generation Laboratory-Based Scanners and ANATOMIX Beamline Microtomography

**Fig. 5:**
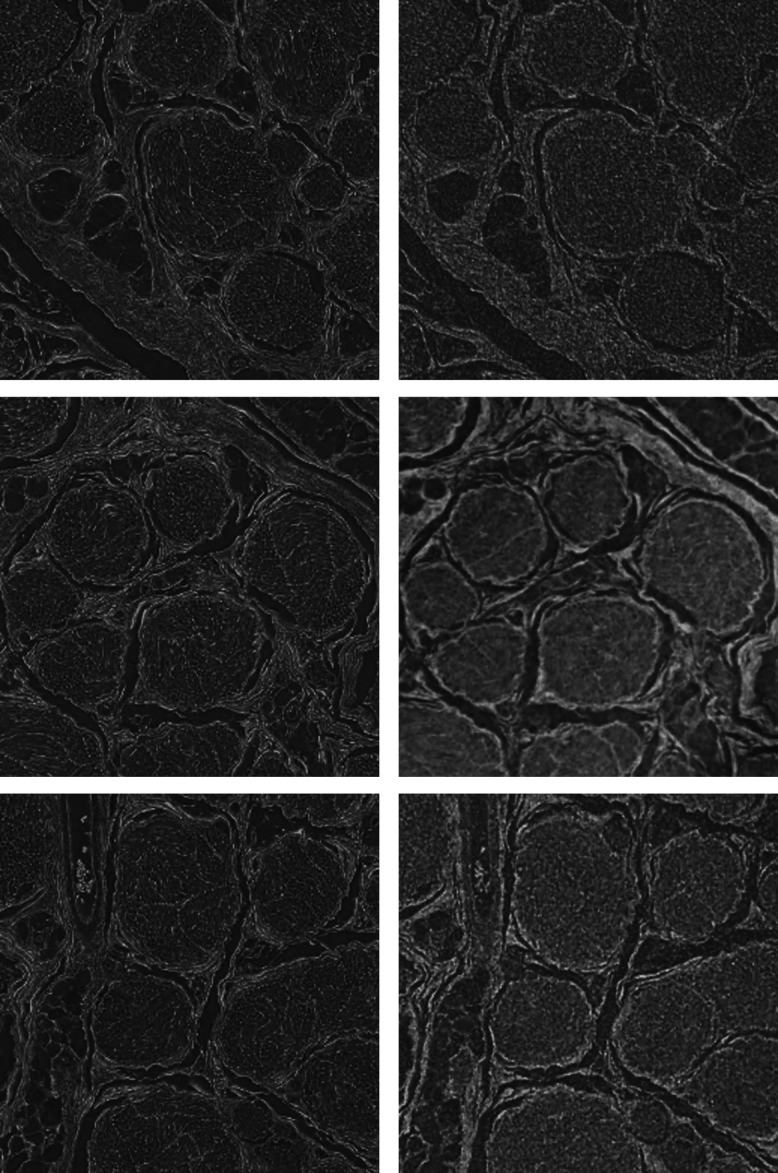
A still image of Video 2 showing the scroll-through 160 cross-sectional virtual histology slices, prepared by the software VGStudio MAX, of the porcine nerve obtained at the ANATOMIX beamline (left column) and registered local tomography data of SkyScan 2214, Exciscope, and Xradia 620 Versa (right column from top to bottom). The spiral structure of the primary nerve fiber bundles is recognized via rotation during scrolling. The width of each box equals about 780  μm (Video 2, MP4, 11503 KB [URL: https://doi.org/10.1117/1.JMI.9.3.031507.2]).).

**Fig. 6:**
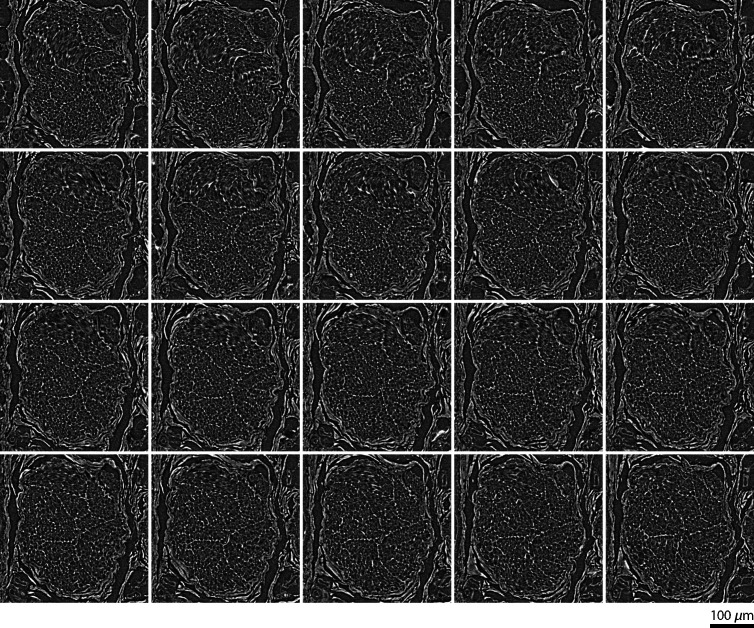
The sequence of 20 virtual slices from the dataset recorded at the ANATOMIX beamline signifies the spiral structure of the selected nerve fiber bundle (counterclockwise).

Similar to tooth imaging, a paraffin-embedded nerve with a diameter of 6 mm does not fit into the field-of-view of the microtomography setups at the spatial resolution selected. Therefore, mosaic-style acquisition^66^ was also applied for porcine nerve imaging at the ANATOMIX beamline. Again, local tomograms from the cutting-edge laboratory-based instruments were registered to data acquired at the ANATOMIX beamline. Figure 5 shows parts of the registered data comparing the gold standard from the ANATOMIX beamline with the selected cutting-edge laboratory-based systems SkyScan 2214, Exciscope, and Xradia 620 Versa, respectively. In the movies, one clearly recognizes a spiral structure to the nerve fiber bundles, which can be approximated by right-handed or left-handed helices with a pitch of about 830  μm. To provide an idea to the readers without access to the movies, Fig. 6 shows a sequence of 20 virtual slices indicating this spiral structure. This representation of the data from the ANATOMIX beamline, however, is much less convincing than scrolling through the series of adjacent slices in Fig. 5.

#### 3.6.1 Performance of SkyScan 2214

It is not really surprising that data from the laboratory-based system show much more noise that the ones from the synchrotron radiation facility, as easily explained by the photon statistics.

The myelin sheaths are slightly more visible in tomography data from the SkyScan 2214 instrument than for the Xradia 620 Versa, as demonstrated by Fig. 5, cp. right column movies at top and bottom.

#### 3.6.2 Performance of Exciscope

Since Exciscope is equipped with a liquid metal source, the photon flux is much higher than for the other two laboratory-based tomography devices. Thus, the density resolution is superior, as seen by the well-preserved myelin sheaths in Fig. 5, second row, right part. This phenomenon is even enhanced by implemented phase retrieval, and consequently, the spiral structure is far more visible. It should be noted that there are right- and left-handed spirals.

#### 3.6.3 Performance of Xradia 620 Versa

Figure 5 (bottom row) clearly demonstrates the substantial improvement of Xradia 620 Versa imaging with respect to the SkyScan 1275 system (cp. Fig. 1). Nevertheless, the myelin sheaths were barely resolved. Regardless, one can detect the spiral structure of the nerve fiber bundles.

### 3.7 Nerve—Clinical Application

High-density resolution and sub-micrometer spatial resolution are required to quantify changes in axonal bundles and myelin sheaths as the result of nerve injury and regeneration. We showed that the cutting-edge tomography systems SkyScan 2214, Exciscope, and Xradia 620 Versa yield enough spatial and density resolution to enable the visibility of several myelin sheaths and axonal bundles. Therefore, x-ray virtual histology, combined with bioengineering, could facilitate protocols for stepwise de- and re-cellularization, including follow-up laboratory imaging for fast feedback control. Already, laboratory-based μCT, with or without the combination of conventional histology, can assist in studies of nerve regeneration, including grafting.^45^^,^^68^ Furthermore, μCT could support the urgent diagnostic need of Wegener’s polyangiitis,^69^ the gold standard of which is histological examination of tissue biopsy.^41^ Moreover, μCT allows for the further investigation of pathomechanisms of this vasculitis and other neurovascular diseases via the computational extraction of neurovascular networks,^70^ or supporting the 3D prospective mapping of neuron connectivity, to understand the anatomical context.^71^ Based on the image quality demonstrated herein, we expect the further integration of advanced μCT into the study of nerve disease, injury, and regeneration.

In Fig. 5, the spiral shape of nerve bundles is observed as one scrolls through the reconstructed slices. This behavior has been described previously as a chevron pattern in longitudinal histotomographical nerve slices.^43^^,^^44^ Whilst the function of this pattern warrants further investigation, one explanation could be a gain in stability, analogous to (steel) wire ropes.^72^

### 3.8 Limitations of the Study

The comparative study of next-generation tomography systems for virtual histology has several drawbacks, as already pointed out by manufacturers during data acquisition. The 36-h limit was chosen to create comparable and fair conditions between the laboratory systems as well as to address the real-life lab operation at an imaging platform. Longer scanning times would probably yield improved results considering the reference images from the synchrotron radiation facilities were acquired with a much higher photon flux. The present study did not consider the unequal numbers of photons used for the individual tomograms.

Measurements with the SkyScan 2214 system in March 2021 did not achieve expected data quality; therefore, the measurements were repeated in July 2021, which might have resulted in some bias in terms of direct comparison.

At the time of the measurements (March 2021), the Exciscope system was still in a prototype stage. Therefore, the optics only allowed for data acquisition with an effective pixel size of 1.3  μm. Simply exchanging a standard optical element would allow for pixel sizes of 0.65  μm. Since the experiments, Exciscope has designed and built a system with improved specifications. This system has an entirely rebuilt mechanical platform complete with vibration dampening, temperature control, redesigned radiation shielding, electrical control, and safety systems. Furthermore, the system has now a motion system with seven motorized axes, which allows for improved control and automation through the software interface. The vertical object stage is ready to make 280-mm helical CTs with a few microns’ resolution. In addition, the rotation stage has a typical error motion of only 0.5 to 0.6  μm, which together with a high-resolution detector will allow for an isotropic spatial resolution of 1  μm.

The lack of the PhaseEvolve software for the Xradia 620 Versa system at the stage of data analysis, and the limited pixel size of the Exciscope system, made a direct comparison of the CNR impossible. Together with spatial resolution, CNR is an essential metric in assessing the image quality of acquired tomograms.^56^

Spatial resolution depends not only on the pixel size employed, but also on many other parameters such as the mechanical stability of the entire system and the design of the detection unit. The spatial resolution, we have calculated here, should be treated with care, because the analysis of the power spectrum of reconstructions used^63^^,^^73^ is challenging and less straightforward than, for example, the measurement of test patterns. Nonetheless, the quantities derived with the model provided by Mizutani et al.^63^ are consistent with visual inspection.

### 3.9 User-Friendliness

The main author, a master’s student in medicine, together with the second author, an experienced physicist and an expert in the field, observed the 36-h measurements at the three manufacturers of the cutting-edge, laboratory-based microtomography systems. During the setup of the experiments, the software for the x-ray microscopes was explained in detail. The user-friendliness of the software was compared on the basis of (i) intuitive interface, (ii) structural organization, (iii) efficiency and effectiveness, and (iv) reliability.

Overall, the operation by a novice was best supported by the Xradia 620 Versa software. This interface allowed for a rather simple and intuitive measurement setup. Proceeding through the well-structured software required user input, which was assisted by a compact two-page manual. A live camera showed the sample position within the instrument. Time saving and precise sample centering were based on double-clicking on radiographs at 0 deg and 90 deg rotation angles. A single click allowed for repositioning the sample along the x axis, y axis, and z axis as well as the source and detector along a single axis. Fragile samples were protected from collisions by automatically measuring the sample diameter. Filter and source settings could be comfortably modified via software. A table in the short two-page overview assisted in choosing the best suitable filter, and rapid pre-scanning enabled scouting. Subsequent fine-tune sample centering was performed automatically after manual center identification in the pre-scan data. In addition, “recipes” could be created to facilitate a session for scanning multiple samples. The estimated scanning time was met.

Bruker’s software for SkyScan 2214 was also user-friendly. The interface, however, was less self-explanatory. It consisted of one screen combining visualization of the sample using a camera, an immense live-view x-ray window, and the necessary settings. This approach was advantageous for experienced users, but a novice might miss essential adjustments, due to the absence of guidance throughout the preparation steps. Additional settings were hidden in the “options” panel. The sample could be rotated by 360 deg and relocated in the x, y, and z directions, automatically. Translation in the x and y dimensions was simplified by drag-and-drop. Additionally, the detector could be mechanically moved in one dimension. Automatic sample protection was unavailable, but the source and filter could be chosen comfortably via software. The selection of these settings, however, was not guided and linked to the user’s prior experience with measurements. Similar to Xradia 620 Versa, rapid pre-scanning allowed for scouting, whilst fine-tune centering was performed manually. Finally, estimated scanning time was generally reliable, although it once underestimated the required scan time.

The user-friendliness of Exciscope could not be assessed, since the prototype was missing a user interface in March 2021, when the measurements took place. The prototype required numerous modifications that precluded operation by a novice user. The reliability of the estimated scan time was satisfactory.

In summary, the software of the Xradia 620 Versa convinced the novice user in terms of ease of use. Bruker’s software for SkyScan 2214 offered an almost similarly satisfactory interface, albeit with less simplicity, and user-friendliness could not be assessed for the Exciscope prototype.

The assessment of the usability of the tomography systems was restricted to one novice user, which is a limitation of the present study in consequence of the constraints during the COVID-19 pandemic.

### 3.10 Laboratory-Based Phase Tomography

The present study belongs to the very few comparative experimental approaches to microtomography^13^^,^^14^^,^^16^ and should support the purchase of next-generation, laboratory-based systems, especially for the field of medicine. The time is now ripe to combine absorption and phase tomography in a single laboratory-based device and reach an isotropic spatial resolution close to 1  μm, maybe even to 100 nm and below. The gap between the tomography setups at the synchrotron radiation facilities and the established laboratory-based microtomography systems is becoming more and more narrow.

The volumetric evaluation of tissues of human and animal origin is currently mainly done by serial sectioning, staining, and microscopic imaging of individual slices. This approach has its drawbacks, though, such as restricted spatial resolution perpendicular to the slices, and the numerous preparation artifacts. High-resolution hard x-ray tomography can complement histology. Prior to sectioning, the stained and unstained tissues can be made visible in absorption and phase contrast modes, to extract local densities. Histotomography similarly facilitates decision-making in terms of the cutting location and the angle of a probe for further histological examination.^74^ Similarly, laboratory-based phase tomography can support choosing a region of interest prior to valuable beamtimes at synchrotron facilities.

The zebrafish larva is an appropriate example through which to demonstrate complementary information from the absorption and phase contrast modes (see Fig. 2). The combination of 3D data, using a bivariant histogram, can allow for dedicated segmentation tasks.^75^

### 3.11 Outlook for Virtual Histology

This work demonstrates that the latest laboratory-based systems employing phase and absorption contrast provide scans with the adequate contrast and resolution to differentiate single cells. Previous work has shown that microtomography has excellent correlation with the conventional histology of embedded tissue slices,^14^^,^^24^^,^^26^^,^^74^ and so x-ray microtomography can be referred to as virtual histology. This imaging technique is also compatible with prior MRI-techniques, e.g., diffusion MRI,^76^ or subsequent electron microscopy.^77^ Furthermore, volumetric virtual histology provides a third dimension for histopathological investigation that has typically relied on irreversible, sparse, two-dimensional sectioning. Microtomography does not require stained samples and avoids slicing them, which results in irreversible destruction of the sample. The conventional approach has further drawbacks, including preparation artifacts such as tears, folds, and non-uniform strains, as well as a requirement for staining.

Laboratory-based systems have the potential to enable the faster availability of virtual histology, as it is currently carried out in synchrotron radiation facilities with limited user access—and most often at a great distance from hospitals. Therefore, further potential benefits of the laboratory-based technique should be explored. While it finds broad application in many research areas, as shown in this study, microtomography could complement future clinical work.

The gold standard for histopathological investigation is conventional histology, based on two-dimensional sectioning, staining, and imaging with optical microscopy. This approach runs the risk of missing important areas or of being time-intensive for the pathologist searching for the region of interest in large samples. Therefore, certain tissues could be examined beforehand, using a laboratory-based system to locate relevant areas and define the best sectioning angle,^74^ and to help removing unreasonable samples early.^77^ This will support that subsequent histology shows the relevant areas under the microscope.

The extent to which virtual histology could support pathology as a diagnostic tool in the future needs to be investigated. Open questions could revolve around the effectiveness of pre-scans using microtomography. Scouting might reduce the overall diagnostic time in larger tissue samples. Nevertheless, a rapid scan followed by slicing needs sufficient spatial resolution and contrast in a reasonable measurement time to indicate a slicing position. The question here arises as to what an appropriate time might be. If one plans first to perform a high-resolution scan of the tissue, subsequent histology might not be necessary because of virtual histology’s recently established and validated value for histopathological analysis. However, staining and histology may remain the gold standard in certain cases, e.g., a recent study has shown that Tau proteins in human brains of Alzheimer’s disease patients remain undetected in virtual histology.^13^ Still, for many cases, virtual histology could serve as the sole diagnostic tool with scan-times of approximately 10 h per probe. Future studies should determine and validate label-free virtual histology as a reliable diagnostic tool.

## 4 Conclusions

Due to the advances in instrumentation over the last decade, propagation-based phase-contrast microtomography is no longer reserved for synchrotron radiation facilities. Laboratory x-ray microscopes can provide satisfying (sub-) cellular resolutions to visualize anatomical features with true micrometer resolution. The compared scanners will be further complemented, but they can already provide high-quality datasets of medically relevant hard and soft tissue specimens. These laboratory-based systems not only support beamtime planning by a priori non-destructive isotropic overview scans, but they can also be used in stand-alone imaging. Such imaging can be applied for pre-clinical trials to quantify pathological mechanisms through the versatile zebrafish larva model, the analysis of TCA in mammals, as well as pre-clinical and clinical trials on the regeneration and grafting of nerves.

## References

[1] Schulz G. Deyhle H. Müller B., Imaging the Human Body: Micro-and Nanostructure of Human Tissues, Springer (2012).

[2] Osterwalder M. et al., “Three-dimensional x-ray microscopy of zebrafish larvae,” Proc. SPIE 11586, 115860J (2021). PSISDG 0277-786X 10.1117/12.2583639

[3] Mani-Caplazi G. et al., “Imaging of the human tooth cementum ultrastructure of archeological teeth, using hard x-ray microtomography to determine age-at-death and stress periods,” Proc. SPIE 10391, 103911C (2017). PSISDG 0277-786X 10.1117/12.2276148

[4] Bikis C. et al., “Three-dimensional and non-destructive characterization of nerves inside conduits using laboratory-based micro computed tomography,” J. Neurosci. Methods 294, 59–66 (2018). JNMEDT 0165-0270 10.1016/j.jneumeth.2017.11.005 29129635

[5] Töpperwien M. et al., “Three-dimensional virtual histology of human cerebellum by x-ray phase-contrast tomography,” Proc. Natl. Acad. Sci. U. S. A. 115(27), 6940–6945 (2018). 10.1073/pnas.1801678115 29915047 PMC6142271

[6] Momose A., “Recent advances in x-ray phase imaging,” Jpn. J. Appl. Phys. 44(9R), 6355 (2005). 10.1143/JJAP.44.6355

[7] Paganin D., Coherent X-Ray Optics, Oxford University Press on Demand (2006).

[8] Bravin A. Coan P. Suortti P., “X-ray phase-contrast imaging: from pre-clinical applications towards clinics,” Phys. Med. Biol. 58(1), R1 (2012). PHMBA7 0031-9155 10.1088/0031-9155/58/1/R1 23220766

[r9] Endrizzi M., “X-ray phase-contrast imaging,” Nucl. Instrum. Methods Phys. Res. Sect. A: Accel., Spectrom., Detectors Assoc. Equip. 878, 88–98 (2018). 10.1016/j.nima.2017.07.036

[r10] Khimchenko A. et al., “Implementation of a double-grating interferometer for phase-contrast computed tomography in a conventional system nanotom^®^ m,” APL Bioeng. 2(1), 016106 (2018). 10.1063/1.5022184 31069291 PMC6481705

[r11] Lang S. et al., “Experimental comparison of grating-and propagation-based hard x-ray phase tomography of soft tissue,” J. Appl. Phys. 116(15), 154903 (2014). JAPIAU 0021-8979 10.1063/1.4897225

[12] Zanette I. et al., “Holotomography versus x-ray grating interferometry: a comparative study,” Appl. Phys. Lett. 103(24), 244105 (2013). APPLAB 0003-6951 10.1063/1.4848595

[13] Töpperwien M. et al., “Correlative x-ray phase-contrast tomography and histology of human brain tissue affected by Alzheimer’s disease,” NeuroImage 210, 116523 (2020). NEIMEF 1053-8119 10.1016/j.neuroimage.2020.116523 31935519

[14] Khimchenko A. et al., “Extending two-dimensional histology into the third dimension through conventional micro computed tomography,” NeuroImage 139, 26–36 (2016). NEIMEF 1053-8119 10.1016/j.neuroimage.2016.06.005 27321044

[15] Khimchenko A. et al., “Hard x-ray nanoholotomography: large-scale, label-free, 3D neuroimaging beyond optical limit,” Adv. Sci. 5(6), 1700694 (2018). 10.1002/advs.201700694 PMC6010902 29938163

[16] Drews S. et al., “Comparative micro computed tomography study of a vertebral body,” Proc. SPIE 7078, 70780C (2008). PSISDG 0277-786X 10.1117/12.793815

[17] Bidola P. et al., “Application of sensitive, high-resolution imaging at a commercial lab-based x-ray micro-CT system using propagation-based phase retrieval,” J. Microsc. 266(2), 211–220 (2017). JMICAR 0022-2720 10.1111/jmi.12530 28181677

[18] Krenkel M. et al., “Propagation-based phase-contrast tomography for high-resolution lung imaging with laboratory sources,” AIP Adv. 6(3), 035007 (2016). AAIDBI 2158-3226 10.1063/1.4943898

[19] Töpperwien M. et al., “Three-dimensional mouse brain cytoarchitecture revealed by laboratory-based x-ray phase-contrast tomography,” Sci. Rep. 7, 42847 (2017). SRCEC3 2045-2322 10.1038/srep42847 28240235 PMC5327439

[20] Howe K. et al., “The zebrafish reference genome sequence and its relationship to the human genome,” Nature 496(7446), 498–503 (2013). 10.1038/nature12111 23594743 PMC3703927

[21] McCampbell K. K. Wingert R. A., “New tides: using zebrafish to study renal regeneration,” Transl. Res. 163(2), 109–122 (2014). 10.1016/j.trsl.2013.10.003 24183931 PMC3946610

[22] White R. M. et al., “Transparent adult zebrafish as a tool for in vivo transplantation analysis,” Cell Stem Cell 2(2), 183–189 (2008). 10.1016/j.stem.2007.11.002 18371439 PMC2292119

[23] Babaei F. et al., “Contrast-enhanced x-ray micro-computed tomography as a versatile method for anatomical studies of adult zebrafish,” Zebrafish 13(4), 310–316 (2016). 10.1089/zeb.2016.1245 27058023

[24] Vågberg W. et al., “X-ray phase-contrast tomography for high-spatial-resolution zebrafish muscle imaging,” Sci. Rep. 5, 16625 (2015). SRCEC3 2045-2322 10.1038/srep16625 26564785 PMC4643221

[25] Cörek E. et al., “Shedding light on metal-based nanoparticles in zebrafish by computed tomography with micrometer resolution,” Small 16(31), 2000746 (2020). SMALBC 1613-6810 10.1002/smll.202000746 32567135

[26] Ding Y. et al., “Computational 3D histological phenotyping of whole zebrafish by x-ray histotomography,” Elife 8, e44898 (2019). 10.7554/eLife.44898 31063133 PMC6559789

[27] Osterwalder M. et al., “Hard x-ray microtomography of zebrafish larvae,” Proc. SPIE 11886, 1188614 (2021). PSISDG 0277-786X 10.1117/12.2593119

[28] Bosshardt D. D. Selvig K. A., “Dental cementum: the dynamic tissue covering of the root,” Periodontology 2000 13(1), 41–75 (1997). 10.1111/j.1600-0757.1997.tb00095.x 9567923

[29] Yamamoto T. et al., “Histology of human cementum: its structure, function, and development,” Jpn. Dent. Sci. Rev. 52(3), 63–74 (2016). 10.1016/j.jdsr.2016.04.002 28408958 PMC5390338

[30] Mani-Caplazi G. et al., “Measuring incremental line width and appearance in the tooth cementum of recent and archaeological human teeth to identify irregularities: first insights using a standardized protocol,” Int. J. Paleopathol. 27, 24–37 (2019). 10.1016/j.ijpp.2019.07.003 31550620

[31] Tanner C. et al., “Extended-field synchrotron microtomography for non-destructive analysis of incremental lines in archeological human teeth cementum,” Proc. SPIE 11840, 1184019 (2021). PSISDG 0277-786X 10.1117/12.2595180

[32] Naji S. et al., “Cementochronology, to cut or not to cut?” Int. J. Paleopathol. 15, 113–119 (2016). 10.1016/j.ijpp.2014.05.003 29539545

[33] Lieberman D. E., “Life history variables preserved in dental cementum microstructure,” Science 261(5125), 1162–1164 (1993). SCIEAS 0036-8075 10.1126/science.8356448 8356448

[34] Colard T. et al., “Toward the adoption of cementochronology in forensic context,” Int. J. Legal Med. 132(4), 1117–1124 (2018). IJLMEA 1427-1596 10.1007/s00414-015-1172-8 25773917

[35] Cerrito P. et al., “Parturitions, menopause and other physiological stressors are recorded in dental cementum microstructure,” Sci. Rep. 10, 5381 (2020). SRCEC3 2045-2322 10.1038/s41598-020-62177-7 32214148 PMC7096390

[36] Bertrand B. et al., “Age at death estimation by cementochronology: too precise to be true or too precise to be accurate?” Am. J. Phys. Anthropol. 169(3), 464–481 (2019). 10.1002/ajpa.23849 31049939

[37] Le Cabec A. et al., “Nondestructive adult age at death estimation: visualizing cementum annulations in a known age historical human assemblage using synchrotron x-ray microtomography,” Am. J. Phys. Anthropol. 168(1), 25–44 (2019). 10.1002/ajpa.23702 30431648

[38] Lee S. K. Wolfe S. W., “Peripheral nerve injury and repair,” J. Am. Acad. Orthop. Surg. 8(4), 243–252 (2000). 10.5435/00124635-200007000-00005 10951113

[39] Pan D. Mackinnon S. E. Wood M. D., “Advances in the repair of segmental nerve injuries and trends in reconstruction,” Muscle Nerve 61(6), 726–739 (2020). 10.1002/mus.26797 31883129 PMC7230025

[40] Lubetzki C. et al., “Remyelination in multiple sclerosis: from basic science to clinical translation,” Lancet Neurol. 19(8), 678–688 (2020). 10.1016/S1474-4422(20)30140-X 32702337

[41] Gross W. Trabandt A. Reinhold-Keller E., “Diagnosis and evaluation of vasculitis,” Rheumatology 39(3), 245–252 (2000). RHEUBD 0080-2727 10.1093/rheumatology/39.3.245 10788531

[42] Bikis C. et al., “Three-dimensional imaging and analysis of entire peripheral nerves after repair and reconstruction,” J. Neurosci. Methods 295, 37–44 (2018). JNMEDT 0165-0270 10.1016/j.jneumeth.2017.11.015 29179953

[43] Töpperwien M. et al., “Phase-contrast tomography of sciatic nerves: image quality and experimental parameters,” J. Phys.: Conf. Ser. 849, 012001 (2017). JPCSDZ 1742-6588 10.1088/1742-6596/849/1/012001

[44] Bartels M. et al., “Myelinated mouse nerves studied by x-ray phase contrast zoom tomography,” J. Struct. Biol. 192(3), 561–568 (2015). JSBIEM 1047-8477 10.1016/j.jsb.2015.11.001 26546551

[45] Hopkins T. M. et al., “Combining micro-computed tomography with histology to analyze biomedical implants for peripheral nerve repair,” J. Neurosci. Methods 255, 122–130 (2015). JNMEDT 0165-0270 10.1016/j.jneumeth.2015.08.016 26300184 PMC4604061

[46] Lewis J. R., “The system usability scale: past, present, and future,” Int. J. Hum.–Comput. Interact. 34(7), 577–590 (2018). 10.1080/10447318.2018.1455307

[47] Kumar A. A. et al., “An evaluation of the user-friendliness of Bayesian forecasting programs in a clinical setting,” Br. J. Clin. Pharmacol. 85(10), 2436–2441 (2019). BCPHBM 0306-5251 10.1111/bcp.14066 31313335 PMC6783600

[48] Gonzalez-Bermejo J. et al., “Evaluation of the user-friendliness of 11 home mechanical ventilators,” Eur. Respir. J. 27(6), 1236–1243 (2006). 10.1183/09031936.06.00078805 16481386

[49] Salmon P. Liu X. Sasov A., “A post-scan method for correcting artefacts of slow geometry changes during micro-tomographic scans,” J. X-Ray Sci. Technol. 17(2), 161–174 (2009). JXSTE5 0895-3996 10.3233/XST-2009-0220 19696469

[50] Feldkamp L. A. Davis L. C. Kress J. W., “Practical cone-beam algorithm,” J. Opt. Soc. Am. A 1(6), 612–619 (1984). JOAOD6 0740-3232 10.1364/JOSAA.1.000612

[51] Paganin D. M. Gureyev T. E., “Phase contrast, phase retrieval and aberration balancing in shift-invariant linear imaging systems,” Opt. Commun. 281(5), 965–981 (2008). OPCOB8 0030-4018 10.1016/j.optcom.2007.10.097

[52] Paganin D. et al., “Simultaneous phase and amplitude extraction from a single defocused image of a homogeneous object,” J. Microsc. 206(1), 33–40 (2002). JMICAR 0022-2720 10.1046/j.1365-2818.2002.01010.x 12000561

[53] Stampanoni M. et al., “Trends in synchrotron-based tomographic imaging: the SLS experience,” Proc. SPIE 6318, 63180M (2006). PSISDG 0277-786X 10.1117/12.679497

[54] Marone F. Stampanoni M., “Regridding reconstruction algorithm for real-time tomographic imaging,” J. Synchrotron Radiat. 19(6), 1029–1037 (2012). JSYRES 0909-0495 10.1107/S0909049512032864 23093766 PMC3480277

[55] Weitkamp T. et al., “The tomography beamline ANATOMIX at Synchrotron SOLEIL,” J. Phys.: Conf. Ser. 849, 012037 (2017). JPCSDZ 1742-6588 10.1088/1742-6596/849/1/012037

[56] Rodgers G. et al., “Optimizing contrast and spatial resolution in hard x-ray tomography of medically relevant tissues,” Appl. Phys. Lett. 116(2), 023702 (2020). APPLAB 0003-6951 10.1063/1.5133742

[57] Yushkevich P. A. et al., “User-guided 3D active contour segmentation of anatomical structures: significantly improved efficiency and reliability,” NeuroImage 31(3), 1116–1128 (2006). NEIMEF 1053-8119 10.1016/j.neuroimage.2006.01.015 16545965

[58] Klein S. et al., “Elastix: a toolbox for intensity-based medical image registration,” IEEE Trans. Med. Imaging 29(1), 196–205 (2009). ITMID4 0278-0062 10.1109/TMI.2009.2035616 19923044

[59] Shamonin D. P. et al., “Fast parallel image registration on CPU and GPU for diagnostic classification of Alzheimer’s disease,” Front. Neuroinf. 7, 50 (2014). 10.3389/fninf.2013.00050 PMC3893567 24474917

[60] Lehmann T. M. Gonner C. Spitzer K., “Addendum: B-spline interpolation in medical image processing,” IEEE Trans. Med. Imaging 20(7), 660–665 (2001). ITMID4 0278-0062 10.1109/42.932749 11465471

[61] Cheng K. C., “A life-span atlas for the zebrafish,” Zebrafish 1(2), 69–69 (2004). 10.1089/zeb.2004.1.69 18248218

[62] Burrell J. C. et al., “A porcine model of peripheral nerve injury enabling ultra-long regenerative distances: surgical approach, recovery kinetics, and clinical relevance,” Neurosurgery 87(4), 833–846 (2020). NEQUEB 10.1093/neuros/nyaa106 32392341

[63] Mizutani R. et al., “A method for estimating spatial resolution of real image in the Fourier domain,” J. Microsc. 261(1), 57–66 (2016). JMICAR 0022-2720 10.1111/jmi.12315 26444300

[64] Andrew M. et al., “New technologies for x-ray microscopy: phase correction and fully automated deep learning based tomographic reconstruction,” Proc. SPIE 11840, 118400I (2021). PSISDG 0277-786X 10.1117/12.2596592

[65] Santos R. et al., “A comprehensive map of molecular drug targets,” Nat. Rev. Drug Discov. 16(1), 19–34 (2017). NRDDAG 1474-1776 10.1038/nrd.2016.230 27910877 PMC6314433

[66] Vescovi R. et al., “Tomosaic: efficient acquisition and reconstruction of teravoxel tomography data using limited-size synchrotron x-ray beams,” J. Synchrotron Radiat. 25(5), 1478–1489 (2018). JSYRES 0909-0495 10.1107/S1600577518010093 30179188 PMC6140399

[67] Newham E. et al., “A robust, semi-automated approach for counting cementum increments imaged with x-ray computed tomography,” bioRxiv (2021). 10.1371/journal.pone.0249743 PMC8568193 34735460

[68] Heimel P. et al., “Iodine-enhanced micro-CT imaging of soft tissue on the example of peripheral nerve regeneration,” Contrast Media Mol. Imaging 2019, 7483745 (2019). 10.1155/2019/7483745 31049044 PMC6458925

[69] Diamantopoulos A. P. et al., “The fast-track ultrasound clinic for early diagnosis of giant cell arteritis significantly reduces permanent visual impairment: towards a more effective strategy to improve clinical outcome in giant cell arteritis?” Rheumatology 55(1), 66–70 (2016). RHEUBD 0080-2727 10.1093/rheumatology/kev289 26286743

[70] Cao Y. et al., “Synchrotron radiation micro-tomography for high-resolution neurovascular network morphology investigation,” J. Synchrotron Radiat. 26(3), 607–618 (2019). JSYRES 0909-0495 10.1107/S1600577519003060 31074423

[71] Kuan A. T. et al., “Dense neuronal reconstruction through x-ray holographic nano-tomography,” Nat. Neurosci. 23(12), 1637–1643 (2020). NANEFN 1097-6256 10.1038/s41593-020-0704-9 32929244 PMC8354006

[72] Chaplin C., “Failure mechanisms in wire ropes,” Eng. Fail. Anal. 2(1), 45–57 (1995). EFANEM 1350-6307 10.1016/1350-6307(95)00004-A

[73] Modregger P. et al., “Spatial resolution in Bragg-magnified x-ray images as determined by Fourier analysis,” Phys. Status Solidi (a) 204(8), 2746–2752 (2007). 10.1002/pssa.200675685

[74] Stalder A. K. et al., “Combined use of micro computed tomography and histology to evaluate the regenerative capacity of bone grafting materials,” Int. J. Mater. Res. 105(7), 679–691 (2014).1862-5282 10.3139/146.111050

[75] Bikis C. et al., “Sensitivity comparison of absorption and grating-based phase tomography of paraffin-embedded human brain tissue,” Appl. Phys. Lett. 114(8), 083702 (2019). APPLAB 0003-6951 10.1063/1.5085302

[76] Trinkle S. et al., “Synchrotron x-ray micro-CT as a validation dataset for diffusion MRI in whole mouse brain,” Magn. Reson. Med. 86(2), 1067–1076 (2021). MRMEEN 0740-3194 10.1002/mrm.28776 33768633 PMC8076078

[77] Morales A. et al., “Micro-CT scouting for transmission electron microscopy of human tissue specimens,” J. Microsc. 263(1), 113–117 (2016). JMICAR 0022-2720 10.1111/jmi.12385 26854176 PMC4907811

